# Physical Activity, Sedentary Behaviour, and Screen Time Amongst Culturally and Linguistically Diverse Australian Children and Adolescents: A Scoping Review of Quantitative and Qualitative Studies

**DOI:** 10.1002/hpja.70109

**Published:** 2025-10-02

**Authors:** Ana Maria Contardo Ayala, Kate Parker, Natalie Lander, Lauren Arundell, Niamh O'Loughlin, Nicola D. Ridgers, Susan Paudel, Anthony Walsh, Jo Salmon

**Affiliations:** ^1^ Institute for Physical Activity and Nutrition (IPAN) Deakin University Geelong Australia; ^2^ School of Exercise and Nutrition Sciences Deakin University Geelong Australia; ^3^ School of Education Deakin University Geelong Australia; ^4^ School of Health and Social Development Deakin University Geelong Australia; ^5^ Centre for Physical Activity, Sport and Exercise Sciences Coventry University Conventry UK; ^6^ Alliance for Research in Exercise, Nutrition and Activity (ARENA) University of South Australia Adelaide Australia

**Keywords:** adolescent, child, cultural diversity, physical activity, sedentary behaviour

## Abstract

**Objectives:**

This scoping review synthesised evidence on the prevalence of, and factors influencing, physical activity (PA), sedentary behaviour (SB), and screen time (ST) among Culturally and Linguistically Diverse (CALD) children and adolescents in Australia.

**Methods:**

Five databases were searched in May 2025. Eligible studies examined prevalence and factors influencing PA, SB and ST among CALD youth. Quantitative data were summarised descriptively. Qualitative findings were synthesised thematically using the socio‐ecological model.

**Results:**

Twenty‐six studies (11 quantitative, 15 qualitative) were included. Quantitative findings showed that CALD youth generally had lower PA and ST than their peers; some groups reported higher SB. There was diversity across cultures, with each group facing unique factors influencing movement behaviours. PA barriers included cultural and gender norms, academic priorities, safety concerns and limited facilities, while facilitators included PA enjoyment, peer/parental support, and school‐based opportunities. For SB and ST, facilitators of more ST included enjoyment, stress relief, lack of alternatives, social connections and safety concerns, while barriers included parental awareness of harms and imposed restrictions.

**Conclusions:**

CALD youth face culturally specific barriers to increasing PA and reducing SB, with limited research on ST determinants.

## 1 Introduction

Since the first census (1911), the Australian population has experienced a significant increase in cultural and linguistic diversity (CALD), defined by country of birth (i.e., non‐English speaking countries) and/or language spoken at home (i.e., not English), ethnicity, religion and migrant status [1]. Immigrant children living in Australia from low‐ and middle‐income countries (LMIC), along with children from non‐English speaking backgrounds (NESB), consistently have higher odds (e.g., odds ratio range 1.5–1.7 [2]) and are more likely (e.g., prevalence ratio 1.29–1.42 [3]) to live with overweight or obesity compared to their Australian‐born and English‐speaking background (ESB) counterparts. Moreover, immigrants arriving in Australia as children (5–11 years) and adolescents (12–17 years) have significantly higher odds of living with overweight or obesity during adulthood [4]. As such, improving the health of CALD children and adolescents (hereafter, youth) in Australia is crucial.

Promoting healthy waking movement behaviours among youth, which include physical activity (PA), sedentary behaviours (SB) and screen time (ST), is essential for preventing non‐communicable diseases such as cardiometabolic diseases [5, 6, 7, 8]. However, less than a quarter of youth in Australia meet PA and ST recommendations of at least an hour a day of moderate‐to‐vigorous‐intensity PA (MVPA) and less than 2 h/day of recreational ST, respectively [9]. Despite comprising a significant proportion of the Australian population (~31.5%) [10], CALD youth in Australia remain disproportionately underrepresented in movement behaviour research [11, 12]; there is no synthesis of evidence describing if and how adherence to the recommendations varies between different CALD groups. Such information would help identify specific groups at higher risk of sub‐optimal movement behaviour engagement.

Understanding the prevalence, barriers and facilitators of movement behaviours among CALD youth in Australia is particularly important as immigration, sociocultural and environmental factors are unique to each country. Recent reviews have examined movement behaviours among CALD youth, including two focusing on studies from the United States and Europe [11, 13], and two on global immigrant youth [14, 15]. However, no reviews have synthesised evidence of the prevalence and factors influencing movement behaviours among CALD youth in Australia. A review focusing specifically on CALD youth in Australia has the potential to inform future national health policies and strategies to promote waking movement behaviours in this at‐risk population group. Therefore, the aim of this scoping review was to synthesise evidence from quantitative and qualitative studies examining movement behaviours among youth of CALD backgrounds (5–17 years) in Australia. Specifically, we aimed to: (i) describe the prevalence of these behaviours and adherence to national movement guidelines among CALD groups; (ii) examine how these outcomes compare with non‐CALD peers, where data were available; and (iii) identify factors associated with movement behaviours within CALD youth populations.

## 2 Methods

### 2.1 Search Strategy and Article Selection

Five electronic databases (MEDLINE Complete, CINAHL Complete, SPORTDiscus, ERIC and APA PsychInfo) were searched in March 2024 (updated May 2025) using key search terms related to ‘physical activity’, ‘sedentary behaviour’, ‘screen time’, ‘children’, ‘adolescents’, ‘CALD’ and ‘Australia’ (Appendix A). Backward and forward citation tracking was also conducted on articles identified through the database search. The search strategy was developed by AMCA and KP and reviewed by JS and NDR. Articles were imported into Covidence [16] for duplicate removal and screening. Titles, abstracts and full texts were screened for inclusion by two independent reviewers (AMCA and KP), using the inclusion/exclusion criteria (Table 1). Discrepancies were discussed until a consensus was reached, with input from the authorship team where required. There was excellent agreement between reviewers at both the title and abstract (98.6% agreement) and full text (90.24% agreement) screening stages. This scoping review follows the outline and structure of the PRISMA‐ScR checklist.

**TABLE 1:** Eligibility criteria for article selection.

	Criteria
Inclusion criteria	Published since 2000 Published in English language Primary research studies Quantitative and qualitative studies Focused on CALD children and adolescent populations in Australia Peer‐reviewed Has reported either prevalence, correlates, factors influencing (e.g., barriers, facilitators) physical activity (including sport and physical education), sedentary behaviour or screen time
Exclusion criteria	Abstracts, dissertations, conference proceedings, reviews and meta‐analyses, commentaries, editorials or protocol papers Participants with mean age < 5 or > 18 years Studies specifically targeting populations with clinical health conditions (e.g., diabetes, autism, obesity) Studies focused on Aboriginal and Torres Strait Islanders Intervention studies

### 2.2 Data Extraction and Analysis

Data extracted included sample characteristics (e.g., sample size), CALD status indicator (e.g., language spoken at home), non‐CALD participant characteristics (e.g., Anglo‐Australian) and study characteristics (e.g., Australian state/territory where the study was conducted, the study type [quantitative or qualitative], recruitment method, data collection method/s and data analysis). Findings from each study related to either prevalence and/or factors associated (e.g., barriers and facilitators) with PA, SB and ST were also extracted by all authors and checked for reliability by AMCA and KP. Studies included in this review used varying definitions of CALD, including language spoken at home, country/region of birth, religion, migration status and acculturation. To manage this variation in our synthesis, when possible, participants were grouped based on their similar reported backgrounds. Quantitative study groups were *Migrants* (Immigrant children); *African* (Migrants from Sub‐Saharan Africa); *Non‐English‐speaking background; Asian* (Chinese, Southeast [Malaysia, Vietnamese, Cambodian], Asian‐Australian, Vietnamese‐Australian); and *Middle Eastern*. For qualitative studies, the following groups were formed: *Muslim*; *Asian* (i.e., Chinese, Vietnamese, Cambodian); *Middle Eastern*; *Africans* (i.e., South Sudanese and refugees) and immigrants from Africa (i.e., South Sudan, Somalia, Ethiopia); *Migrants*: (a) children from refugee and asylum seeker backgrounds (i.e., Somali, Ethiopian, El Salvadorian); (b) migrant families (i.e., Turkish, Greek, Indian, Chinese); (c) immigrants from Africa, the Middle East, India and Vietnam.

Findings from quantitative studies were synthesised descriptively, and deductive thematic analysis was used to synthesise findings from qualitative studies [17]. Thematic analysis in a scoping review is a process used to identify, analyse and report patterns (themes) within qualitative data. The socio‐ecological model (SEM) [18] was used to categorise factors influencing PA, SB and ST as used in a previous review [19]. These factors were categorised into the following levels: individual, interpersonal, organisational, community and policy by two authors. Any disagreements between authors were resolved through discussion, and where consensus could not be reached, a third author was consulted to make the final decision. The Appraisal Tool for Cross‐Sectional Studies (AXIS tool) [20] and the 10‐item Critical Appraisal Skills Programme (CASP) checklist [21] were used to assess methodological quality for the quantitative and qualitative studies, respectively (Appendix B: Part A). Studies with lower quality scores were included due to the scoping nature of the review, but their limitations were noted and considered when interpreting the findings.

## 3 Results

**FIGURE 1:**
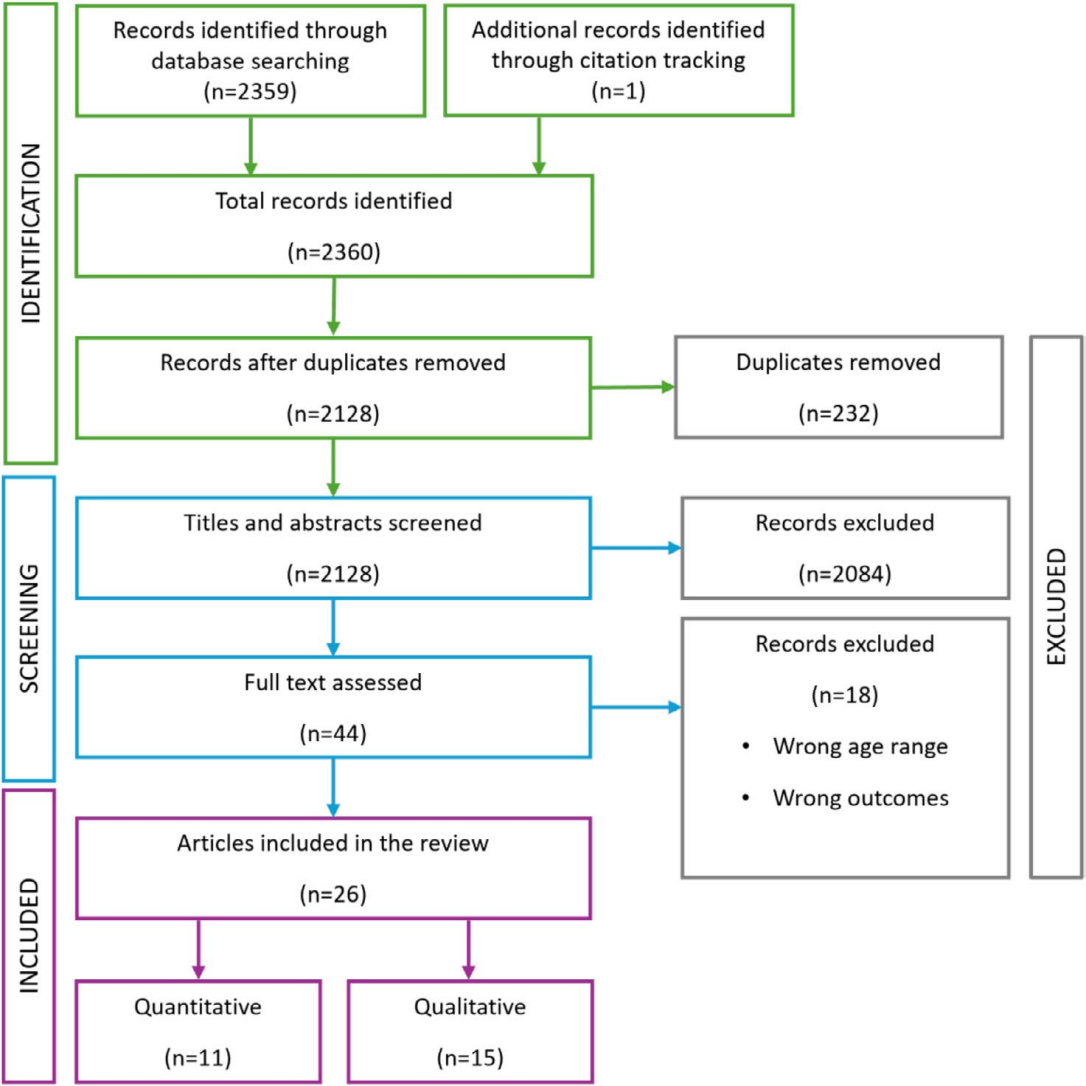
Flow diagram for article selection.

The search retrieved 2360 articles (see Figure 1). Following duplicate removal (*n* = 232), 2128 article titles and abstracts were screened, and 2084 articles were excluded at this stage. Full text screening was conducted for 44 articles, with 18 records excluded due to the wrong exposure/outcome (*n* = 8) or target population (*n* = 10). This resulted in 26 articles (11 quantitative and 15 qualitative) being included. The findings are synthesised separately by quantitative and qualitative studies.

### 3.1 Synthesis of Findings From Quantitative Studies

Table 2 presents the characteristics of the quantitative studies (*n* = 11). Eight different CALD communities were represented, with studies using various CALD indicators, including: (a) being children of immigrants, further separated according to high and low income backgrounds [22, 25, 31]; (b) country of birth such as China [24, 26], Malaysia [26], Cambodia [26], Vietnam [26, 27, 28]; (c) region of birth country such as Asia [26, 29, 30], the Middle East [29], and sub‐Saharan Africa [23]; (d) NESB status [12], and; (e) level of acculturation (Traditional, Integrated, Assimilated, Marginalised) [23]. Across studies, Asian groups (particularly Chinese and Vietnamese) were the most frequently represented, while Middle Eastern, African and refugee populations were examined in only one or two studies each. The studies included a total of 21 820 participants, with 27% (*n* = 5837) from a CALD community. Female and male participants were included in nine studies [12, 22, 23, 24, 25, 26, 29, 30, 31], one study included only females [28] and one study included only males [27]. The behaviours reported were PA (*n* = 8) [12, 22, 23, 24, 26, 27, 28, 29], including sport (*n* = 3) [24, 25, 30, 31], SB (*n* = 3) [22, 29, 31] and ST (*n* = 3) [12, 23, 26]. The methodology used to assess the behaviours and factors associated was mostly self‐report surveys (*n* = 9) [22, 24, 25, 26, 27, 28, 29, 30, 31], and proxy‐report surveys (*n* = 4) [12, 23, 29, 31], with one study utilising a device‐based measure of PA and SB [26]. The methodological quality assessment of quantitative studies is presented in the Appendix B: Part B.

**TABLE 2:** Quantitative studies: Characteristics and summary results by behaviour of interest and sex, when provided.

First author/year—study design (*)	CALD indicator used by the article	*n*	Sex (% female)	Age (years: mean, median or range)	Results				
					Behaviour assessed	Outcome reported	CALD indicator	Results	
Ahmed, Uddin [22]—2022—CS	Immigrant children^a^	Anglo‐Aust = 2260 HIC = 619 LMIC = 343	48.2%	12.4 ± 0.5	Meet PA recs	% achieved	Anglo‐Aust	11.0	
							HIC	11.0	
							LMIC	**6.0**	
					Meet ST recs	% achieved	Anglo‐Aust	27.0	
							HIC	29.0	
							LMIC	**37.0**	
Paudel, Veitch, Mishra—2024 [12]—CS	NESB ESB	NESB = 279 ESB = 3864	NESB = 51.3 ESB = 47.8	7.3 ± 2.9	Meet PA recs	% achieved	NESB	**16.8**	
							ESB	**25.1**	
					Meet ST recs	% achieved	NESB	62.7	
							ESB	58.3	
					Meet PA and ST recs	% achieved	NESB	**9.3**	
							ESB	**14.9**	
Renzaho, Swinburn and Burns [23]—2008—CS	Migrants from Sub‐Saharan Africa	337	Traditional^c^: 43.1% Integrated^c^: 53.1% Assimilated^c^: 46.5% Marginalised^c^: 53.9%	Traditional: 6.5 ± 0.4 Integrated: 7.6 ± 0.2 Assimilated: 9.1 ± 0.4 Marginalised: 6.0 ± 0.2	PA	min/day: mean [95% CI]	Traditional (ref)	94.8 [82.8, 106.9]	
							Integrated	**137.4 [120.5, 154.3]**	
							Assimilated	99.6 [80.1, 119.1]	
							Marginalised	96.9 [85.2, 108.6]	
					SED	min/day: mean [95% CI]	Traditional (ref)	188.6 (166.2, 497.9)	
							Integrated	**241.1 [225.0, 257.3]**	
							Assimilated	**221.2 [192.8, 249.6]**	
							Marginalised	179.0 [157.2, 200.7]	
Chao, Khan, Shi—2025—CS [24]	Chinese	251	57.8	13.31 ± 1.34	Meet PA recs	days/week: mean (±SD)		2.26 ± 1.91	
					Team sports time	hours/week: mean (±SD)		1.29 ± 2.08	
					ST	Hours/day: mean (±SD)		2.06 ± 1.94	
					PE time	hours/week: mean (±SD)		1.03 ± 0.61	
Ahmed, Gomersall, and Khan [25]—2023—LG	Immigrant children^b^	Anglo‐Aust = 2776 HIC = 742 LMIC = 445	48.4%	6.3 ± 0.4	Individual sport	% participate	Aust (ref)	45.4	
							HIC	**41.0**	
							LMIC	**52.3**	
					Team sport	% participate	Aust (ref)	**47**	
							HIC	**41.7**	
							LMIC	**27.2**	
					Meet ST	% achieved	Aust (ref)	14.0	
							HIC	52.2	
							LMIC	49.5	
Strugnell, Renzaho [26]—2015—CS	CHN‐Aust SE‐Asian (Malaysia, Vietnam, Cambodia), Anglo‐Aust	CHN‐Aust = 100 SE‐Asia*n* = 76 Anglo‐Aust = 110	CHN‐Aust = 55% SE‐Asian = 52.6% Anglo‐Aust = 60%	CHN‐Aust = 13.8 (13.1–14.6) SE‐Asian = 13.8 (12.8–14.4) Anglo‐Aust = 14.0 (13.1–15.3)	Sedentary	min/day: mean	Anglo‐Aust (ref)	509	
							CHN‐Aust	**546**	
							SE‐Asian	**546**	
					LPA	min/day: mean	Anglo‐Aust	225	
							CHN‐Aust	**164**	
							SE‐Asian	**170**	
					MVPA	min/day: mean	Anglo‐Aust	24	
							CHN‐Aust	24	
							SE‐Asian	23	
Wilson and Dollman [27]—2007—CS	Viet‐Aust Anglo‐Aust	Viet‐Aust = 62 Anglo‐Aust = 118	0%	13.6 ± 1.0	MVPA (*n* blocks/day)	blocks/day: mean (±SD)	Anglo‐Aust	3.7 ± 2.4	
							Viet‐Aust	3.2 ± 2.4	
					MVPA School day	blocks/day: mean (±SD)	Anglo‐Aust	3.3 ± 2.3	
							Viet‐Aust	2.9 ± 2.3	
					VPA	blocks/day: mean (±SD)	Anglo‐Aust	2.3 ± 2.2	
							Viet‐Aust	1.9 ± 1.7	
					VPA School day	blocks/day: mean (±SD)	Anglo‐Aust	1.9 ± 1.8	
							Viet‐Aust	1.8 ± 1.8	
Wilson, Dollman [28]—2009—CS	Viet‐Aust Anglo‐Aust	Viet‐Aust = 39 Anglo‐Aust = 74	100%	13.9 ± 0.6	MVPA	blocks/day: mean (±SD)	Anglo‐Aust	3.5 ± 3.0	
							Viet‐Aust	2.3 ± 2.6	
					VPA	blocks/day: mean (±SD)	Anglo‐Aust	1.0 ± 2.0	
							Viet‐Aust	0.7 ± 1.9	
								**Boys**	**Girls**
Hardy, King [29]—2013—CS	Middle Eastern (ME) Asian ESB	ME = 280 Asian = 449 ESB = 4169	ME = 49.3% Asian = 51.7% ESB = 49.7	ME = 8.3 ± 2.2 Asian = 8.3 ± 2.2 ESB = 8.4 ± 2.2	Meet PA recs	% achieved	ME/Low SES	14.5	8.4
							ME/Middle SES	5.7	21.9
							ME/High SES	7.1	26.5
							Asian/Low SES	17.3	15.9
							Asian/Middle SES	27.8	13.4
							Asian/High SES	21.2	17.5
					Meet ST recs	% achieved	ME/Low SES	37.4	49.5
							ME/Middle SES	26.5	30
							ME/High SES	32.6	22.1
							Asian/Low SES	50.9	61
							Asian/Middle SES	32.8	62.6
							Asian/High SES	50.6	66.8
Lushington, Wilson [30]—CS	Asian‐Aust	Caucasian‐Aust = 297 Asian‐Aust = 101	39.2%	Caucasian‐Aust: Female = 17 ± 0.6 Male = 16.9 ± 0.6 Asian‐Aust: Female = 17.1 ± 0.7 Male = 17 ± 0.6	Organized sport	min/day: mean	Caucasian‐Aust	286	208
							Asian‐Aust	146	117
					MVPA	min/day: mean	Caucasian‐Aust	252	151
							Asian‐Aust	194	104

*Note:* Bold values denote statistical significance. Note: Complete prevalence data was requested from Zulfiqar et al. (2019); however, the information has not been received as of this date of submission.

Abbreviations: Aust, Australian; CHN, Chinese; ESB, English‐speaking backgrounds; Gen, generation; HIC, high income country; HIC, high‐income country; LIC, low income country; LMIC, low—middle income country; LPA, light intensity physical activity; LSAC, The Longitudinal Study of Australian Children; ME, Middle Eastern; MIC, middle income country; NESB, Non‐English‐speaking background or primarily speaking a non‐English language; PA, physical activity; PE, physical education; SE, South‐East; SES, socioeconomic status; Viet, Vietnamese.

* Study desing: CS: cross‐sectional; LG: longitudinal.

^a^ Children were classified as children of immigrant parents if they were born in Australia and one or more of their parents were born overseas.

^b^ Child's immigrant status was determined by parental (usually mother's) reported birth country of the child, mother and maternal grandparents.

^c^ Traditional = African; Integrated = both; Assimilated = Australian; Marginalised = neither.

#### 3.1.1 Prevalence of Physical Activity, Sedentary Behaviour and Screen Time

CALD youth consistently engaged in less PA than their non‐CALD peers. Only 6% of children from LMIC backgrounds met PA guidelines compared to 11% in Anglo‐Australian and HIC groups [22]. Similarly, just 6% of Middle Eastern boys and 8.4% of girls from low socioeconomic status (SES) backgrounds meet PA recommendations [29]. A significantly lower proportion of children from NESB met PA guidelines (17% vs. 25%, *p* = 0.002) than their ESB peers. Asian‐Australians exhibited lower MVPA levels than Caucasian‐Australians boys (194 vs. 252 min/day) and girls (104 vs. 151 min/day) [30]. Chinese‐Australian adolescents recorded −5 min/day, while South East Asian adolescents spent −7.9 min/day compared to Anglo‐Australians [26]. Vietnamese‐Australian adolescents also demonstrated fewer blocks of MVPA (i.e., 30‐min activity blocks; 2.3–3.2 vs. 3.5–3.7 blocks/day for girls and boys, respectively) and VPA (0.7–1.9 vs. 1.0–2.3 blocks/day) when compared to Anglo‐Australian adolescents [27, 28]. Chinese adolescents engaged in MVPA 2.26 days per week, spent 1.03 h in physical education, 1.29 h in team sports and 0.78 h in non‐team sports per week [24]. Sub‐Saharan African migrant groups reported lower average daily PA, with Traditional (95 min/day), Assimilated (99 min/day) and Marginalised (97 min/day) categories, while Integrated children showed a higher average of 137 min/day [23].

Sport participation was lower among CALD groups. Asian‐Australian adolescents participated in less organised sport than Caucasian‐Australians (146 vs. 286 min/day for boys and 117 vs. 208 min/day for girls) [30], and immigrant children from LMIC backgrounds reported the lowest team sport participation (27.2%) compared to HIC (41.7%) and Anglo‐Australian (47%) peers [25].

Chinese‐Australian and South East‐Asian adolescents recorded more sedentary time (~546 min/day) than Anglo‐Australians (509 min/day) [26]. Assimilated and Integrated African children were the most sedentary (221 and 241.1 min/day, respectively) compared to Marginalised and Traditional (179 and 188 min/day, respectively) [23].

ST adherence differed among CALD groups. More immigrant LMIC children met guidelines (37%) than their HIC (29%) and Anglo‐Australian (27%) peers [22]. Also, one study reported that fewer immigrant LMIC children met screen guidelines (49.5%) than HIC (52.2%) and Anglo‐Australian (54%) children [25]. Similarly, less than half Middle Eastern children met ST guidelines (22%–50%) [22], and less than two‐thirds of Asian children met the guidelines (33%–67%) [29]. Highest levels of ST guidelines achievement were amongst low SES Middle Eastern boys and girls, low SES Asian boys and high SES Asian girls [29]. There was no significant difference in meeting ST guidelines between children from NESB and those from ESB, with 63% of NESB and 58% of ESB children meeting the guidelines [12]. Regarding ST, Chinese adolescents spent 2.06 h of ST per day and used screens 5.2 days per week [24].

#### 3.1.2 Factors Influencing Physical Activity (Including Sport and Physical Education), Sedentary Behaviour and Screen Time

Four of the 11 quantitative studies reported factors influencing PA [12, 27, 28] and sport [25] These were categorised according to the relevant level of the socio‐ecological model. Only individual and interpersonal level factors were identified.

##### 3.1.2.1 Individual Level

Age was inversely associated with meeting PA guidelines among children from both NESB (OR: 0.81, 95% CI: 0.69–0.95) and ESB (OR: 0.85, 95% CI: 0.82–0.87) [12]. Across different CALD groups separated by SES of parent birth country, there were three factors consistently related to lower odds of children participating in team sports. These included being female (CALD LMIC OR: 0.39, 95% CI: 0.32–0.47; CALD HIC OR: 0.40, 95% CI: 0.36–0.47; Australian OR: 0.42, 95% CI: 0.39–0.45), higher ST (CALD LMIC OR: 0.85, 95% CI: 0.76–0.95; CALD HIC OR: 0.80, 95% CI: 0.74–0.86; Australian OR: 0.85, 95% CI: 0.81–0.89), and experiencing more psychological difficulties (CALD LMIC OR: 0.94, 95% CI: 0.93–0.96; CALD HIC OR: 0.95, 95% CI: 0.94–0.96; Australian OR: 0.95, 95% CI: 0.94–0.95). In contrast, higher sleep duration was positively associated with team sport participation among children from HIC (OR: 1.09, 95% CI: 1.02–1.18) and Australia (OR: 1.07, 95% CI: 1.03–1.12) [25].

##### 3.1.2.2 Interpersonal Level

Having a large yard was positively associated with meeting PA (OR: 4.14, 95% CI: 1.72–10.00) and combined guidelines (OR: 4.48, 95% CI: 1.61–12.41) among NESB children [12]. Anglo‐Australian adolescents reported higher parent (3.6 ± 1.4) and peer support (4.9 ± 1.3) for PA than Vietnamese‐Australian boys (2.9 ± 1.3 and 4.4 ± 1.5, respectively) [27]. Parent encouragement, help, and co‐participation were significantly greater among Anglo‐Australian girls [28] and boys [27] compared to Vietnamese‐Australian boys. For example, Anglo‐Australian boys had higher scores for mother help (4.0 ± 1.3 vs. 3.1 ± 1.2), father help (4.3 ± 1.3 vs. 3.3 ± 1.2), and mother plays with (2.7 ± 1.0 vs. 2.3 ± 0.6) [27]. Among girls, Anglo‐Australians also scored higher than Vietnamese‐Australian in mother help (median = 4.0 vs. 3.0), encouragement (5.0 vs. 3.0), and father plays with them (3.0 vs. 2.0) [28]. Best friend support for PA was higher in Anglo‐Australian girls and boys (4.9 ± 1.3) than in Vietnamese‐Australian peers (4.4 ± 1.5), and maternal PA modelling was stronger among Anglo‐Australian boys (2.7 ± 1.0 vs. 2.3 ± 0.6) [28].

### 3.2 Synthesis of Findings From Qualitative Studies

Table 3 presents the characteristics of the included qualitative studies (*n* = 15). A range of CALD communities was represented, including children from immigrant families of various backgrounds (*n* = 3), Africa (*n* = 2), the Middle East (*n* = 4), Asia (*n* = 3), and the Muslim community (*n* = 3). A total of 377 participants were included across the qualitative studies, ranging from 5 [36] to 160 [34] participants. Nine studies included both male and female participants [34, 35, 37, 38, 41, 42, 44, 45, 46], four studies focused on females [36, 39, 40, 43], and one study focused on males [33]. Participants' ages ranged from five to 18 years. Behaviours assessed included PA (*n* = 11) [34, 35, 37, 38, 39, 40, 41, 42, 43, 45, 46] (including sport [*n* = 4] [33, 36, 39, 44] and physical education [*n* = 3] [39, 43, 44]) and ST (*n* = 2) [35, 38]. The methodological quality assessment is presented in Appendix B: Part C.

**TABLE 3:** Characteristics of qualitative studies, by alphabetical order of first author.

First author/year	CALD indicator used by the articles	*n*	Sex (% female)	Age (years: mean, median or range)	Behaviour	Measurement tool
Dennaoui, Kolt [32]—2024	Middle Eastern	27	100%	12–17	PA; Sport	Interviews and focus groups: parents, adolescents
Fox and Paradies [33]—2020	Children from refugee and asylum seeker immigrant backgrounds: Somali, Ethiopia, El Salvador)	10	0%	9–12	Sport	Interviews: school principal, students
Green, Waters [34]—2003	Migrant families: Turkish, Greek, Indian, Chinese	160	50%	Interviews: 5–14 Focus groups: 8–15	PA; Sport	Interviews and focus groups: grandparents, parents, children
Hardy, Hector [35]—2016	Middle Eastern	21	43%	5–12	PA; ST	Interviews: parents
Harwood, Sendall [36]—2021	Muslim	2	100%	12–18	Sport	Ethnography, observation, digital storytelling
Hayba, Khalil, and Allman‐Farinelli [37]—2021	Middle Eastern	32	59%	13–21	PA	Focus groups
Hayba, Shi and Allman‐Farinelli [38]—2021	Middle Eastern	26	Not reported	13–14	PA; ST	Interviews: parents
Knez, Macdonald and Abbott [39]—2012	Muslim	11	100%	14	PA; PE; Sport	Interviews: adolescents
Macdonald, Abbott [40]—2009	Muslim	10	100%	14–16	PA	Interviews: adolescents
Mude and Mwanri [41]—2016	African (South Sudanese)	8	62.5%	Not reported	PA	Interviews: parents
Pang, Macdonald and Hay [42]—2015	Chinese	12	83%	10–15	PA	Interviews: children, adolescents
Pang and Hill [43]—2018	Chinese, Vietnamese, Cambodian	11	100%	12–15	PA; PE; Sport	Interviews: adolescents
Pang and Macdonald [44]—2016	Chinese	12	83%	10–15	PE	Interviews: adolescents, observations
Renzaho, McCabe and Swinburn [45] —2012	Refugees and immigrants from Africa (South Sudan, Somalia, Ethiopia)	14	Interviews: 67% Focus groups: 50%	Interviews: 14.3 Focus groups: 16.8	PA	Interviews and focus groups: parents, adolescents
Renzaho, Green [46]—2018	Immigrants from Africa, the Middle East, India, and Vietnam	48	Not reported	Parents of children aged < 15	PA	Interviews: parents

*Note:* All qualitative studies had a cross‐sectional study design.

Abbreviations: PA, physical activity; PE, physical education; SB, sedentary behaviour; ST, screen time.

#### 3.2.1 Factors Influencing Physical Activity (Including Sport and Physical Education), Sedentary Behaviour and Screen Time

Table 4 provides an overview of the factors influencing PA, SB and ST, by CALD group and framed using the socio‐ecological model. Appendices C and D summarise example quotes.

**TABLE 4:** Perceived factors influencing movement behaviours by CALD group and level of the socio‐ecological model (in order of most to least frequently reported).

	Muslim (*n* = 3 studies)	Asian (*n* = 3 studies)	Middle Eastern (*n* = 4 studies)	Africans (*n* = 2 studies)	Migrants (*n* = 3 studies)
**Individual**					
**Physical activity**					
Enjoyment: Enjoyment of sport/PA	+	+	+		
Preferences: Engaging in preferred sports					+
Preferences: Preference for active travel			+		
Social belonging: Sense of belonging in sport	+				
Body awareness: Achieving a specific body composition			+		
Health awareness: Knowledge of the benefits of PA			+		
Body awareness: Unathletic appearance limits PE opportunities		−			
Competing priorities: External motivations to engage in academic activities over PA		−	−		
Competing priorities: Perceived lack of PA choices	−				
Competing priorities: Screen time displaces time available for PA			−		
Language/cultural barriers: Language barriers leading to exclusion in sport		−			
**Screen time**					
Preferences: Preference for ST despite awareness of excessive engagement			+		
Preferences: ST viewed as a stress reliever			+		
Preferences: Lack of alternative activities			+		
Health awareness: Behavioural issues when ST is limited			+		
Health awareness: Limited knowledge of ST risks			+		
**Interpersonal**					
**Physical activity**					
Parent/family: Parent support (tangible, practical, and emotional)	+	+	+	+	
Parent/family: Co‐participation with family	+	+	+		
Parent/family: Parental role modelling			+	+	
Parent/family: Parent knowledge of the benefits of PA		+	+		
Parent/family: Parent ST restrictions enabling more PA			+		
Peers: Peer support		+	+		
Teachers: Teacher support for school sport participation	+	+			
Development of social capital and new friendships through sport	+				+
Coach: Coach recognition of skills		+			
Social belonging: Sport can bring people together					+
Parent/family: Parental time constraints and lack of motivation for supporting PA	−	−	−	−	
Parent/family: Parental concerns about sport affecting academic success		−	−		
Parent/family: Parent perception that exercise will result in too much weight loss				−	
Teachers: teachers reinforce gender stereotypes		−			
Social barriers: Presence of boys negatively impacts participation for girls	−		−		
Social barriers: Perceived social status requirement for sports inclusion		−			
Social barriers: Gender‐related discomfort in sports participation					−
Cultural norms: that females should do housework, cook, get ready for marriage, etc. limit sport participation	−		−		
Cultural norms: limit physical touch or interaction between males and females	−		−		
Cultural norms: that engaging in PA and sport is not ladylike and is unacceptable behaviour			−		
Cultural norms: that boys are more capable than girls limit girls' PE participation		−			
Cultural norms: to wear hijab and dress modestly limiting sport participation			−		
Cultural norms: to be ‘white’ limits participation in outdoor PA		−			
Cultural norms: in children's independence for outdoor play				−	
Financial resources: Economic investment in other pursuits instead of sport		−			
**Screen time**					
Social benefit: ST allows social interactions			+		
Parent/family: Parental knowledge of the negative effects of ST			**−**		
Parent/family: Parents imposed restrictions and rules to limit ST			**−**		
**Organisational level**					
**Physical activity**					
School: School cultural inclusion/adaptation (clothing/uniforms to enable sport participation)	+		+		
School: School opportunities for participation in a variety of sports and PA	+		+		
School: Gender‐specific support at schools (single‐sex classes facilitate greater PE participation for girls)	+				
School: School lunchtime opportunity for outdoor active play			+		
Sport clubs: Provision of transport by sports clubs					+
School: Homework load reducing time available for PA			**−**		
School: Lack of access or facilities at school			**−**		
School: School lack of PA opportunities for girls			**−**		
School: School prioritizes academic achievement over PA/sport			**−**		
**Community level**					
**Physical activity**					
Safety: Safety concerns		**−**	**−**	**−**	**−**
Environmental resources: Lack of access to places to be active			**−**	**−**	
Environmental resources: Lack of transport				**−**	**−**
Environmental resources: Lack of interesting facilities for PA		**−**			
Environmental resources: Technology limits active travel				**−**	
Equity support: Perceived need for more equitable support/funding for sport participation			**−**		
**Screen time**					
Safety: Safety concerns					+
**Public policy level**					
**Physical activity**					
Policy barriers: Australian policy prohibits leaving children unattended outdoors— limits outdoor PA				**−**	
Policy barriers: Complex legislation on the use of public space limits PA participation				**−**	

*Note:* Symbols: + = facilitator; − = barrier.

Abbreviations: PA = physical activity; PE = physical education; ST = screen time.

##### 3.2.1.1 Individual Level

There was little consistency in reported individual‐level barriers and facilitators to PA participation between CALD groups. Enjoyment of sport (Muslim, Asian and Middle Eastern) [36, 37, 43, 44] and PA (Muslim and Middle Eastern) [35, 37, 40] were reported as the most common individual‐level facilitators, mostly among adolescents. Other facilitators included having a preference for participating in certain sports (migrants) [34] and active travel (Middle‐Eastern) [35], feeling a sense of belonging in sport (Muslim) [36], desiring a certain body composition perceived as achievable with PA (Middle‐Eastern) [37], and knowing the importance of PA for physical and mental benefits (Middle‐Eastern) [37].

Barriers included language barriers resulting in exclusion from participation, and external and conflicting motivations to engage in academic activities over sport and PA (particularly Chinese [42] and Middle‐Eastern [32] girls). A perceived lack of choice in physical education (Muslim adolescent females) [39] having an unathletic appearance (Asian adolescent females) [44] and ST displacing time available for PA (Middle‐Eastern) [38] were additional individual‐level barriers.

A range of individual SB and ST barriers and facilitators was mentioned by parents of Middle‐Eastern children aged 5–12 years [35]. These included preferring SB despite awareness of sometimes engaging in excessive amounts, viewing ST as a stress reliever, having a lack of alternative activities, behavioural issues when ST is limited, and lacking knowledge of the detrimental effects of excessive ST [35].

##### 3.2.1.2 Interpersonal Level

Muslim adolescents [36] and children of immigrant parents [33] mentioned the development of social networks and new friendships as facilitators of sports participation. A range of social factors was perceived to positively influence both sport and PA participation across all CALD groups, including peer [32, 37, 44] and parent support [32, 35, 38, 40, 42], parental role modelling [38, 45], family co‐participation [32, 39, 43] and parental knowledge of the benefits of PA [35, 38]. Additionally, teacher support for school sports participation was reported as a facilitator by adolescents from both Muslim [39] and Asian backgrounds [43]. Other, less commonly reported interpersonal facilitators included sport bringing people of various backgrounds together (migrants) [33], parent‐imposed ST restrictions and rules enabling more PA (Middle‐Eastern) [35], and coaches recognising and praising sporting skills (Asian) [44].

Cultural stereotypes were seen as barriers to sport and PA participation, mostly affecting girls; however, the specific stereotypes differed between cultures and religions [32, 36, 39, 40, 42, 43, 44, 45, 46]. Muslim and Middle‐Eastern female adolescents felt they were expected to spend their time doing housework, cooking and preparing for marriage (being ‘ladylike’) and dressing modestly, and the cultural norm to limit interactions between males and females made participation in certain types of PA challenging (e.g., dancing) [32, 36, 39, 40]. A cultural preference for lighter skin tones and concerns about sport affecting academic success limited PA among Chinese youth [42, 43, 44]. African parents reported concerns that exercise would result in too much weight loss, which contradicts their cultural belief that a larger body size is a sign of prosperity and good health [45]. Dennaoui, Kolt [32] also reported that parents view sport as an embarrassing and unacceptable thing for their daughters to participate in. Other common interpersonal barriers were the inability to afford fees associated with sports (migrants from Africa, the Middle East, India and Vietnam) [33, 41, 46] and parental lack of time and motivation to facilitate participation in sport and PA among youth across multiple CALD groups (Muslim, Asian, Middle‐Eastern, and African) [35, 39, 41, 42, 43].

Middle‐Eastern adolescents [37] and parents [38] viewed ST as a tool for social interactions in two separate studies. Despite this, Middle‐Eastern parents also understood the negative effects of ST and imposed rules to limit this at times [38].

##### 3.2.1.3 Organisational Level

At the organisational level, the only facilitator mentioned by multiple CALD groups was school, which offered opportunities for participation in a variety of PA (Muslim and Middle‐Eastern adolescents) [32, 37, 39]. Additional school offerings that led to more PA were the provision of single‐sex classes (Muslim) [36], scheduled lunchtimes enabling outdoor active play (Middle‐Eastern) [35], and allowances to alter clothing to suit cultural norms and enable sports participation (Muslim) [32, 39]. Additionally, Hayba, Khalil, and Allman‐Farinelli [37] described how Middle‐Eastern adolescents stated that their schools assisted them in limiting the amount of ST they engage in. Sports clubs providing transport to and from practice and games enabled participation among migrant children that otherwise would not have been possible [33].

A number of organisational level barriers to PA (including sport and physical education) were reported by Middle‐Eastern adolescents [37] and parents [38] including lack of access to sports facilities when at school, lack of opportunities for girls and older students to be active at school, schools prioritising academic achievement over being active, and high homework loads reducing the time available for sport and PA.

##### 3.2.1.4 Community Level

Lack of access to places to be active in the community (Middle‐Eastern and African) [35, 41], lack of transport (African) [41], and safety concerns (Asian, Middle‐Eastern, Chinese, African and migrants from Africa) [37, 41, 42, 45, 46] were commonly reported community‐level barriers to participation in PA and sports. South Sudanese expressed concerns about neighbourhood safety [41] and reported that migrant parents from Africa, the Middle East, India, and Vietnam are leading to their children spending too much time on screens indoors [46]. Asian adolescents reported a lack of interesting facilities for PA [42]. Middle‐Eastern parents spoke of the need for more equitable support and funding for sport participation [38], and African parents saw technology as something that displaced the need for active travel and exercise [41].

##### 3.2.1.5 Public Policy Level

African parents perceived Australian policies that prohibit leaving children outdoors unattended [41] and the complexity of legislation regarding the use of public spaces [45] as barriers to outdoor PA opportunities for youth. Parents also reported that these policies may lead to more ST [41].

## 4 Discussion

### 4.1 Summary of Key Findings

This scoping review synthesised quantitative and qualitative evidence regarding the prevalence of, and factors associated with PA (including sport and physical education), SB and ST amongst CALD youth in Australia. Among studies reporting prevalence, CALD youth were less likely to meet PA guidelines and engaged in a lower volume of PA compared to non‐CALD youth. While ST levels varied, some CALD groups exhibited lower ST than their non‐CALD counterparts [12, 22, 23, 25]. Barriers to PA were primarily identified at the individual and interpersonal levels, including personal preferences, cultural norms, societal gender expectations, and family priorities, with only two studies reporting on policy‐level influences. For SB and ST, facilitators of more ST at the individual level included enjoyment, stress relief, lack of alternatives, and limited awareness of harms; at the interpersonal level, social connections; at the community level, safety concerns were commonly reported. Barriers were mainly identified at the interpersonal level, including parental knowledge of harms and the imposition of restrictions or rules. The findings highlight that CALD youth are a key group for targeted PA promotion strategies and programs, as they are generally less active than their Australian‐born peers and have culturally specific barriers and facilitators to target.

### 4.2 Prevalence of Movement Behaviours

Despite the limited number of studies and the heterogeneity in methodologies, CALD youth in Australia tended to be less active and participated in less sport than their Australian‐born peers, a trend consistent with findings from immigrant children in Europe and the United States [13]. While CALD youth were generally less active than their non‐CALD peers, findings for ST were more mixed, with some CALD groups reporting lower levels of screen use, also aligning with previous research [12]. These differences may reflect variations in family expectations (e.g., prioritisation of academic study over leisure screen use), or cultural values that emphasise alternative activities such as household or community responsibilities. Such findings highlight the heterogeneity within CALD populations and the need to avoid treating CALD youth as a homogenous group. Instead, future studies should examine subgroup‐specific determinants of screen use to better inform targeted interventions.

### 4.3 Factors Associated With Movement Behaviours

A range of barriers and facilitators in relation to PA, SB and ST were identified, with some common to all youth, regardless of CALD background. For example, participation in activities they enjoy [29, 39], parent support and co‐participation with family [47] were consistent facilitators of PA among both CALD and non‐CALD youth. Similarly, safety concerns [48] and cost [48] are common barriers reported among CALD groups internationally. However, some factors appeared unique to CALD youth in Australia. For instance, language barriers, perceived social status for sport inclusion, and cultural norms to be ‘white’, particularly among Asian families, often led to PA being deprioritised [42, 44]. Academic pressure emerged as a particularly strong barrier for those from Asian backgrounds. This likely reflects cultural values that prioritise educational attainment (e.g., via extra homework or tutoring) as a pathway to future security and upward mobility, particularly for migrant families seeking to establish themselves in Australia. Additionally, cultural expectations and gender norms varied across CALD groups, shaping PA, SB, and ST differently depending on the specific background [32, 39]. These findings highlight the need for culturally responsive strategies to address the unique barriers CALD groups face.

### 4.4 Implications for Schools and Community Programs

Schools, in particular, can play a key role by fostering inclusive PA environments. This could involve teacher/peer‐led support [49], challenging gender stereotypes [50], offering a variety of enjoyable PA options (i.e., whole of school approach) [51], providing single‐sex PE classes, creating PA opportunities during school breaks, and balancing academic demands with PA opportunities. Additionally, schools can strengthen engagement by partnering with key CALD community groups to co‐design and deliver culturally relevant PA initiatives and increase awareness of their role among CALD‐specific barriers/facilitators.

A recent systematic review of culturally adapted PA interventions targeting CALD youth demonstrated that only four of the 20 interventions (*n* = 2 from Australia) were successful at increasing PA in the target population [52]. These successful interventions used cultural adaptations including bilingual staff/facilitator, delivered in the language of the participants, met cultural expectations, engaged with stakeholders, and used participatory approaches (formative work, focus groups) [52]. Further high‐quality research is required to identify effective means (e.g., cultural adaptation programs/interventions) to increase PA and reduce SB and ST in CALD youth.

### 4.5 Methodological Limitations

There was significant heterogeneity in study samples, CALD indicators, and assessment methods across the included studies, making it challenging to synthesise results and draw definitive conclusions. For example, types of CALD populations varied across studies (e.g., children of immigrant parents, refugees, specific religious groups), as did comparison groups (e.g., CALD vs. non‐CALD or between CALD subgroups). Additionally, CALD status was inconsistently defined using indicators such as country of birth, language spoken, religious affiliation, refugee status, and acculturation level. These challenges are common in research on Australia's CALD population groups [53] and are also observed internationally [1] highlighting the need for a more standardised approach to defining and reporting on CALD populations. Moreover, most studies examined PA when SB and ST are also key movement behaviours that warrant further exploration.

Additionally, most studies relied on cross‐sectional and self‐reported data, which are subject to recall and reporting biases. Small sample sizes in several studies, along with the underrepresentation of certain CALD groups (e.g., India and the Philippines, which are in the top five countries of birth in Australia) [54], and the low quality of several of the included studies (Appendix B: Part B and Part C) further constrain the ability to draw definitive conclusions. Additionally, while this review synthesised all available literature, the high variability underscores the importance of future research focusing on specific CALD subgroups. Furthermore, few studies reported community or public involvement in the research process, limiting the inference of relevance and cultural responsiveness of the findings. Tailored investigations into distinct cultural or linguistic groups will allow for more precise insights and the development of effective, culturally responsive interventions.

### 4.6 Practical Implications and Future Research

Future research should adopt clearer, more consistent reporting of CALD indicators, using frameworks from government and health agencies. Standardising key demographic and migration‐related variables would improve comparability and targeted recommendations. Given the variability in CALD classifications, research should also focus on specific cultural or linguistic subgroups for more precise insights and culturally responsive interventions. Culturally appropriate PA, SB, and ST opportunities are needed to address individual and structural barriers. For example, program delivery in multiple languages or by bilingual staff can reduce communication barriers and increase trust. Co‐design with CALD community leaders and families ensures programs reflect community priorities and values. Gender‐specific activity options, such as single‐sex PE or sport sessions, may help address cultural and religious norms that restrict mixed‐gender participation. Flexibility in program scheduling to avoid clashes with cultural or religious practices (e.g., prayer times, festivals) can also support engagement. Beyond schools, community‐based initiatives led by councils, faith‐based groups or clubs can offer culturally safe spaces and improve family engagement. Policy measures, such as subsidising sport costs or requiring equity‐focused reporting, are important for reducing access barriers. Feasibility should be considered: low‐cost or subsidised programs, partnerships with local organisations, and use of existing facilities can enhance sustainability. Longitudinal research on PA, SB, and ST among CALD youth is essential for understanding the effects of acculturation.

## 5 Conclusion

CALD youth in Australia face complex and multifaceted barriers to engagement in movement behaviours, influenced by individual, interpersonal, organisational, community, and public policy factors. While certain facilitators exist, addressing disparities requires a comprehensive, culturally tailored approach that brings together schools, families, and communities. Future studies to inform the development of tailored interventions should prioritise culturally inclusive policies and targeted strategies to ensure equitable opportunities for movement behaviours among CALD youth.

## Ethics Statement

The authors have nothing to report.

## Consent

The authors have nothing to report.

## Conflicts of Interest

The authors declare no conflicts of interest.

## Data Availability

The data that support the findings of this study are available on request from the corresponding author. The data are not publicly available due to privacy or ethical restrictions.

## Appendix A Example Search

**Table:**

Search number	Search terms
1	‘sedentary behavi*’ OR sedentary OR sitting OR ‘sitting time’ OR ‘physical activity’ OR ‘moderate to vigorous physical activity’ OR ‘light intensity physical activity’ OR ‘MVPA’ OR ‘resistance training’ OR ‘strength training’ OR ‘weight training’ OR ‘resistance exercise’ OR exercis* OR sport* OR ‘active recreation’ OR ‘leisure activities’ OR ‘active travel’ OR ‘active transport’ OR cycling OR walking OR running OR ‘active commuting’ OR ‘screen? time’ OR TV OR television OR computer OR laptop OR screen OR smartphone OR ‘cell phone’ OR ‘mobile phone’ OR tablet OR ‘video gam*’ OR ‘electronic gam*’ OR gaming OR ‘social media’ OR ‘digital tablet’
2	cultur* or multicultur* or cultur* divers* or CALD or ‘non‐english speak*’ or NESB or ‘culturally and linguistically diverse’ or migrant* or immigrant* or refugee*
3	Australia* or Victoria or ‘New South Wales’ or Tasmania or Queensland or ‘Northern Territory’ or ‘Western Australia’ or ‘South Australia’ or ‘Australian Capital Territory’
4	adolescen* OR teen* OR youth OR ‘school age*’ OR ‘child*’ OR ‘kid*’ OR ‘young people’
5	1 and 2 and 3 and 4

## Appendix B

- Methodological Quality Assessment

The Appraisal Tool for Cross‐Sectional Studies (AXIS tool) [20] and the 10‐item Critical Appraisal Skills Programme (CASP) checklist [21] were used to assess methodological quality for quantitative and qualitative studies, respectively. Agreement between the two authors (AMC and KP) who completed the method quality assessment for the quantitative studies was high (AXIS: 88.89% agreement, Cohen's kappa = 0.73; CASP: 89.29% agreement, Cohen's kappa = 0.74), and where discrepancies occurred, discussions were conducted with AMC and KP to reach a consensus.

B Methodological Quality Assessment Results for Quantitative Studies

Table B1 provides an overview of the methodological quality of the included quantitative studies. All studies provided a clear definition of their target population and statistical significance and precision estimates, and the results appeared internally consistent and presented according to the described analyses in all studies. Few studies justified the sample size (*n* = 2). No studies provided information regarding non‐responders (*n*=0), and few reported measures undertaken to address and categorise non‐responders (*n* = 3).

**TABLE B1:** Methodological quality of quantitative studies (AXIS tool).

First author	Aims/objectives of the study clear	Study design appropriate for the stated aim(s)	Sample size justified	Target/reference population clearly defined	Sample frame taken from an appropriate population base	Selection process likely to select representative subjects/participants	Measures undertaken to address and categorise non‐responders	Risk factor and outcome variables measured appropriate to study aims	Risk factor and outcome variables measured correctly	Clear what was used to determine statistical significance and/or precision estimates	Methods sufficiently described to enable them to be repeated
Ahmed, Gomersall, and Khan [25]	Yes	Yes	No	Yes	Yes	Yes	No	Yes	Yes	Yes	Yes
Ahmed, Uddin [22]	Yes	Yes	No	Yes	Yes	Yes	No	Yes	Yes	Yes	Yes
Hardy, King [29]	Yes	Yes	No	Yes	Yes	Yes	Do not know	Yes	Yes	Yes	Yes
Lushington, Wilson [30]	Yes	Yes	No	Yes	No	No	Do not know	Yes	No	Yes	Yes
Renzaho, Swinburn, and Burns [23]	Yes	Yes	No	Yes	Yes	No	Yes	Yes	Yes	Yes	Yes
Zulfiqar, Strazdins [31]	Yes	Yes	Yes	Yes	Yes	Yes	Yes	Yes	Yes	Yes	Yes
Strugnell, Renzaho [26]	Yes	Yes	Yes	Yes	No	Yes	Yes	Yes	Yes	Yes	Yes
Wilson, Dollman [28]	No	Do not know	No	Yes	No	No	No	Do not know	Yes	Yes	No
Wilson and Dollman [27]	Yes	Yes	No	Yes	No	No	No	Yes	Yes	Yes	Yes

First author	Basic data adequately described	Response rate raises concerns about non‐response bias	Where appropriate, information about non‐responders described	Results internally consistent	Results for the analyses described in the methods, presented	Authors' discussions and conclusions justified by the results	Limitations of the study discussed	Funding sources or conflicts of interest that may affect the authors' interpretation of the results	Ethical approval or consent of participants attained
Ahmed, Gomersall, and Khan [25]	Yes	Yes	No	Yes	Yes	Yes	Yes	No	Yes
Ahmed, Uddin [22]	Yes	Do not know	No	Yes	Yes	Yes	Yes	No	Yes
Hardy, King [29]	Yes	No	No	Yes	Yes	Yes	Yes	Do not know	Yes
Lushington, Wilson [30]	Yes	Do not know	No	Yes	Yes	Yes	Yes	Do not know	Yes
Renzaho, Swinburn, and Burns [23]	Yes	Do not know	No	Yes	Yes	Yes	Yes	No	Yes
Zulfiqar, Strazdins [31]	Yes	No	No	Yes	Yes	Yes	Yes	No	Yes
Strugnell, Renzaho [26]	Yes	Yes	No	Yes	Yes	Yes	Yes	No	Yes
Wilson and Dollman [27]	No	No	No	Yes	Yes	Yes	No	No	Yes
Wilson, Dollman [28]	No	No	No	Yes	Yes	Yes	No	Do not know	No

C Methodological Quality Assessment Results for Qualitative Studies

Table B2 synthesizes the methodological quality of the included qualitative studies. All studies were found to be valuable in understanding this research field, with only one study being unclear regarding the appropriateness of the qualitative methodology. The main methodological quality issues were regarding the recruitment strategies not appropriate for the aims (*n* = 7) and lack of adequate consideration of the researcher‐participant relationship (*n* = 9). A clear statement of the research aims and research findings, use of an appropriate research design, and consideration of potential ethical issues were common, with all but two studies meeting these criteria.

**TABLE B2:** Methodological quality of qualitative studies (Critical Appraisal Skills Programme).

Author	Clear statement of the aims of the research	Qualitative methodology appropriate	Research design appropriate to address the aims	Recruitment strategy appropriate to the aims	Data collected in a way that addressed the research issue	Relationship between researcher and participant adequately considered	Ethical issues taken into consideration	Data analysis sufficiently rigorous	Clear statement of findings	How valuable is the research?
Fox and Paradies [33]	Yes	Yes	Yes	Yes	Yes	Can't tell	Can't tell	No	Yes	Valuable
Green, Waters [34]	Yes	Yes	Yes	Yes	Yes	No	Yes	No	Yes	Valuable
Hardy, Hector [35]	Yes	Yes	Yes	No	Yes	No	Yes	Yes	Yes	Valuable
Harwood, Sendall [36]	No	Can't tell	Yes	No	Yes	Yes	Yes	Yes	Yes	Valuable
Hayba, Khalil, and Allman‐Farinelli [37]	Yes	Yes	Yes	Yes	Yes	No	Yes	Yes	Yes	Valuable
Hayba, Shi, and Allman‐Farinelli [38]	Yes	Yes	No	Yes	Yes	No	Yes	Yes	Yes	Valuable
Knez, Macdonald, and Abbott [39]	No	Yes	Yes	No	Yes	Can't tell	Yes	No	No	Valuable
Macdonald, Abbott [40]	Yes	Yes	No	No	No	No	No	Can't tell	No	Valuable
Mude and Mwanri [41]	Yes	Yes	Yes	Yes	Yes	No	Yes	Yes	Yes	Valuable
Pang, Macdonald, and Hay [42]	Yes	Yes	Yes	No	No	No	Yes	Yes	Yes	Valuable
Pang and Hill [43]	Yes	Yes	Yes	No	Yes	No	Yes	No	Yes	Valuable
Pang and Macdonald [44]	Yes	Yes	Yes	No	No	No	Yes	Yes	Yes	Valuable
Renzaho, McCabe, and Swinburn [45]	Yes	Yes	Yes	Yes	Yes	Yes	Yes	Yes	Yes	Valuable
Renzaho, Green [46]	Yes	Yes	Yes	Yes	Yes	Yes	Yes	Yes	Yes	Valuable

## Appendix C Qualitative Results: Factors Influencing Physical Activity, Sport and Physical Education by Level of the Socio‐Ecological Model

**Table:**

	Mu	As	M‐E	Af	Mi	An example quote when was provided
*Individual level*						
Enjoyment of sport/PA	Ad; ♀ Ad; ♀	Ad; ♀, ♂	Ad; ♀, ♂ Ch, Ad; ♀, ♂			‘… I just love playing netball (interschool)’ (Muslim, adolescent, female) [40] ‘For me, it's (table‐tennis) just for fun. I do it every Saturday night and Sunday night’ (Asian, adolescent, female) [43] ‘… I do (perform) Indian dancing at functions … you just get up and like have fun’ (Muslim, adolescent, female) [40]
Engaging in preferred sports					Ch, Ad; ♀, ♂	‘We decide which sport we should play. If we see an advertisement in the paper about sports, we show it to our parents and if we like they say it is OK’ (child of migrant parents, male) [34] ‘I am sensible enough to choose the type of sport I like. I decide what I like to play, and my parents let me do it’ (adolescent child of migrant parents, male) [34]
Preference for active travel			Ch; ♀, ♂			
Sense of belonging in sport	Ad; ♀					‘Feel like I belong on the soccer field; no one cares about what I am wearing. I feel comfortable there’ (Muslim, adolescent, female) [36]
Achieving a specific body composition			Ad; ♀			‘I saw a celebrity having a nice body … but you can't just get it. You have to … engage in PA activity, eat well’ (Middle‐Eastern, adolescent, female) [37]
Knowledge of the benefits of PA activity			Ad; ♀, ♂			‘Keeping healthy and training is good. It just makes you feel better’ (Middle‐Eastern, adolescent, male) [37] ‘I think … it helps me deal with my anxiety … just like a bit of jumping about or just playing basketball with my brothers … helps me ease my mind and stress’ (Middle‐Eastern, adolescent, female) [37]
External motivations to engage in academic activities over PA		Ad; ♀	Ad; ♀			‘It [education] gives her more opportunities. More opportunities in the future. I wasn't educated personally. I love the job I do, but then I look at people who are educated, the jobs that they have, I want better for my girls. Studying would be number one’ (Middle‐Eastern, parent) [32]
Language barrier leading to exclusion in sport		Ad; ♀, ♂				‘… since your English is not good enough, you won't know what they're saying, but when your English gets better … you hear stuff … they'll say you're Asian and you can't play sport’ (Chinese, adolescent, male) [44]
Perceived lack of PA choices	Ad; ♀					‘I don't do HPE because I don't like the things they do, so I just bring a note (from home)’ (Muslim, adolescent, female) [39]
Screen time displaces time available for PA			Ad; ♀, ♂			‘When they're sitting on the X‐box all day. There is no exercise; the only exercise they do is their voicebox’ (Middle‐Eastern parent) [38]
Unathletic appearance limits opportunities in PE		Ad; ♀				‘Well, when I was skinny, I just look(ed) so fragile that I'll be pushed over by wind, and now when I'm not that skinny, people just assume that I don't do much exercise anyways … they just don't pick me first’ (Chinese, adolescent, female) [44]
*Interpersonal level*						
Development of social capital and new friendships through sport	Ad; ♀				Ch; ♂	‘The turning point was learning T‐ball. As I learnt with team sport, you need people around to achieve. For the first time in a long time, I was truly happy. I made some friends, gained my self‐confidence and, more importantly, learnt my self‐worth’ (Muslim, adolescent, female) [36]
Sport can bring people together					Ch; ♂	‘On the weekend, the boys are all out playing footy or soccer … and [at school] they play together. So, it doesn't matter, once again, what colour you are … Sport is the glue that holds them together and makes the race go away’ (Principal of male CALD students) [33]
Peer support		Ad; ♀, ♂	Ad; ♀, ♂			‘At camp … we were going on the flying fox, and I'm really scared of heights … but then my classmates, they were all at the end, they're all cheering for me, and telling me that it's ok and I can do it. They're really supportive’ (Chinese, adolescent, female) [44]
Presence of boys negatively impacts participation for girls	Ad; ♀		Ad; ♀			‘When I was playing with the girls, I was having fun, you know, cause I can get the ball and do whatever I like with it, but when the boys come when they mix with you, they are so f*cken selfish, they don't, and they act as if they are the best. I'm too scared because I'm afraid someone might kick me in the leg or something’ (Muslim, adolescent, female) [39] ‘When you're scarfed you can't touch a guy or anything … We have things you should do and things you shouldn't’ (Middle‐Eastern, adolescent, female) [32]
Perceived social status requirement for sports inclusion		Ad; ♂				‘Sometimes, it makes me feel that I have to be popular to be included in sport. … some of them are not even that good at sport, they're just good at socialising, but then there're people who are pretty good at sport, and be like tall and fit, fast and strong, and things like that’ (Chinese, adolescent, male) [44]
Parent support (tangible, practical, and emotional)	Ad; ♀	Ad; ♂	Ch, Ad; ♀, ♂	Ad; ♀		‘I got my weight bench, my dad bought me one, coz I'm like skinny’ (Chinese, adolescent, male) [42] ‘Mummy encourages me to exercise so I do not turn out like herself, I think, because she turned out like, fat. But my mummy is not worried; she is cool for me to exercise’ (African, adolescent, female) [45]
Parental role modelling			Ad; ♀, ♂	Ad; ♀		‘… my husband is very sporty as well. So, he's very active and my kids see that.… he goes to the gym, he plays soccer, he does all this sort of stuff, and they see that. And I love the fact that they say that he's very active. So that kind of encourages them as well’ (Middle‐Eastern parent) [38]
Co‐participation with family	Ad; ♀	Ad; ♀	Ad; ♀, ♂			‘Usually, me and my (older) sister we usually go for a jog at the park, and I'll take a soccer ball, or basketball and we start playing with it’ (Muslim, adolescent, female) [39] ‘… I play with my uncle and aunty. Like we'll have a barbeque we'll set up the volleyball court, we do that probably twice a month’ (Chinese, adolescent, female) [43] ‘My brother plays basketball, soccer and goes to gym … my father is going with me to gym’ (Middle‐Eastern, adolescent, female) [32]
Parent knowledge of the benefits of PA		Ad; ♀	Ch, Ad; ♀, ♂			‘It's [PA] important to me because basically I want him to be healthy’ (Middle‐Eastern parent) [35] ‘I reckon if they can get like two hours a day. That'd be amazing, like two hours of physically being outside, walking, running, being in the fresh air, like filling their lungs with like literally like fresh air, socialising, like skateboarding, swimming, whatever, like two hours a day. Phenomenal’ (Middle‐Eastern parent) [38]
Parent screen time restrictions enabling more PA			Ch, Ad; ♀, ♂			‘By the time they finished their homework. Then they can choose between maybe 10 min computer or whatever they're doing, but the rest have to get outside and jump and play’ (Middle‐Eastern parent) [35]
Parent perception that exercise will result in too much weight loss				Ad; ♀, ♂		‘Sometimes I want my child to put on a little bit of weight, but on the other hand he is very active. … But I think being very active prevents him from putting on weight. I have asked him to reduce his level of activity’ (African parent) [45]
Parental concerns about sport affecting academic success		Ad; ♀				‘My parents are like whenever I ask to join a new sport, they're like ‘What's the point of that? Are you going to get world champion? Are you going to get number 1 like gold medal in sailing? If not, I don't think you should do it, coz it's not going to get you any money and it's not going to get you an OP1 (highest school leavers academic score)’’ (Chinese, adolescent, female) [42]
Parental time constraints and lack of motivation for supporting PA	Ad; ♀	Ad; ♀, ♂	Ch; ♀, ♂	Ch; ♀, ♂		‘He used to attend soccer. Unfortunately, we've had to scrap that this year … Because I couldn't keep up. It was really hard because I was the one who had to take him to training, take him to the matches. I'd have to drag off the other two kids with me and it was just a bit hard’ (Middle‐Eastern parent) [35]
Coach recognition of skills		Ad; ♂				‘The guy who tells us what to do in boxing, he's always like complimenting me being good at techniques’ (Chinese, adolescent, male) [44]
Teacher support for school sport participation	Ad; ♀	Ad; ♀				’…my HPE teacher goes, ‘You're not the best person in sports’ and I agree, but she goes, ‘You always participate and that's what great’, so that's why I have a go’ (Muslim, adolescent, female) [39]
Teachers reinforce gender stereotypes		Ad; ♀				‘In HPE class, not many girls participate in it, coz like we're girls, we don't want to do it, and the teachers is like, you can walk around the field and do nothing’ (Chinese, adolescent, female) [44]
Cultural belief that females should do housework, cook, get ready for marriage etc. limits sport participation	Ad; ♀		Ad; ♀			‘Being a young girl from a Somalia background, it was hard for me to play any sport. The stereotype in Somalia and many other countries is that, if you're a girl, you're meant to stay home and do the housework until you get married’ (Muslim, adolescent, female) [36] ‘Sport is not part of my culture. Girls have cultural expectations, and these are to cook, clean, look after the house and babysit. My aunt said, you can [do] this sport thing at school. After that, it's not on’ (Muslim, adolescent, female) [36] ‘You're a girl. You're more helpful at home than going out and playing sport for someone. You could be at home helping your parents, doing your homework, studying for your exams’ (Middle‐Eastern, adolescent, female) [32]
Cultural belief that boys are more capable than girls limits girls' PE participation		Ad; ♀				‘I guess in PE, the captain chooses the boys first, like, they are the stronger players, and he kind of chooses the girls last. … Some of the girls are strong as well, but coz the boys always want those who's able to kick and able to goal’ (Chinese, adolescent, female) [44]
Cultural norm to wear hijab and dress modestly limits sport participation			Ad; ♀			‘Say an Iranian girl wants to play netball, but the parents see what the netball uniform is, it's like this short little dress, they'll say automatically no, you're showing too much. Or rugby, you have to play in shorts and a T‐shirt. Or basketball it's a singlet and shorts’. (Middle‐Eastern, adolescent, female) [32] ‘With a hijab, you can't wear particular type clothing so it—and if you wear something … [that makes you] different from the team the team looks at you. People look at you, other team members, family, families and parents look at you like, oh my god she's wearing a completely different uniform to the whole team’ (Middle‐Eastern parent) [32]
Cultural norm to be ‘white’ limits participation in outdoor PA		Ad; ♀				‘All I got was getting tanner and tanner in my sport and my dad starts complaining, like oh my gosh, you look like you're getting darker, no no no! They prefer me to be Whiter’ (Chinese, adolescent, female) [42]
Cultural norms limit physical touch or interaction between males and females	Ad; ♀		Ad; ♀			‘If it was girls, okay, but there's boys there and dancing in front of boys is sort of haram, it depends how you are dancing. Like last Wednesday, we were doing line dancing. The first part of the dancing was linking hands with boys, walking around holding hands; me and Alana didn't do it. But some other Muslim boys did, and I was staring at them, I was so shocked, because what you have to do is hold hands, and link arms, I'm not going to do that with boys’ (Muslim, adolescent, female) [39] ‘In our religion and in our culture, it's gender separation. Men are with men and ladies are with ladies and there should be no contact, no seeing each other … it's that cultural barrier that stops us from even having a conversation with … another male’ (Middle‐Eastern, adolescent, female) [32]
Cultural belief that sport is not ladylike and is unacceptable behaviour			Ad; ♀			‘When it comes to marriage, if people want to ask for your daughter's hand, they're going to be embarrassed to tell them … she plays sport’ (Middle‐Eastern parent) [32]
Gender‐related discomfort in sports participation					Ad; ♀	‘…it is hard for our girls to go out to sports clubs to play along with the boys outside…they cannot wear same sports clothes like other girls and cannot play freely with boys around…’ (Migrant parent) [46] ‘…the girls don't know how to play all these Western games…they feel scared and shy to ask male teachers how to use these sports equipment and articles for playing…they feel better if women teachers are there…’ (Migrant parent) [46]
Cultural norms in children's independence for outdoor play				Ch; ♀, ♂		‘You will be remaining inside and thinking my kids are playing outside and you don't even go to check on them, for them here [in Australia] is really bad. You have to go with them, stay in the park with them, and for those who are playing soccer, you put them in the car and take them in the soccer place, and when the soccer finish you come with them back home’ (South Sudanese parent) [41]
Financial beliefs: Economic investment in other pursuits instead of sport		Ad; ♀				
Financial barriers: Inability to afford memberships/fees				Ch; ♀, ♂	Ch, Ad; ♀, ♂	‘… [For a] poor person here [in Australia], the gym really needs money’ (South Sudanese parent) [41] ‘…at this point when we have other expenses like rent, transport costs, food and clothes it is too much to enrol children in sports clubs now…’ (Migrant parent) [46]
*Organisational level*						
Schools cultural Inclusion/Adaptation (clothing/uniforms to enable sport participation)	Ad; ♀					
Gender‐specific support at schools (Single‐sex classes facilitate greater PE participation for girls)	Ad; ♀					
School opportunities for participation in a variety of sports and PA	Ad; ♀		Ad; ♀, ♂			‘On Wednesdays we do sports. We like have a range of sports that you want to do, so you choose, so I chose soccer’ (Muslim, adolescent, female) [39]
School lunchtime opportunity for outdoor active play			Ch; ♀, ♂			‘I'm glad he has a school now that he has a lunchtime playtime. So, I think that kind of makes up for the playtime outside time’ (Middle‐Eastern parent) [35]
Homework load reducing time available for PA			Ch, Ad; ♀, ♂			‘The reason he's not active every day is because of study overload … Takes over your life’ (Middle‐Eastern parent) [35]
Provision of transport by sports clubs					Ch; ♂	
Lack of access to sports facilities at school			Ad; ♀, ♂			‘My school has three big fields, but we're not allowed to be on them lunch and recess’ (Middle‐Eastern, adolescent, female) [37]
School's lack of PA opportunities for girls			Ad; ♀, ♂			‘We have the facilities, but I feel like they restrict us a bit because I go to an all‐girls school … although they, vouch for equality and fairness and like being equal with boys. … they don't really allow us to do physical sports’ (Middle‐Eastern, adolescent, female) [37]
School prioritises academic achievement over PA and sport			Ad; ♀, ♂			‘I don't think there's enough encouragement to be active and to be involved in sport. I really think that Muslim schools need to look at that more carefully because there's such a focus on academics. I think that kind of gets left behind’ (Middle‐Eastern parent) [38]
*Community level*						
Lack of access to places to be active			Ch; ♀, ♂	Ch; ♀, ♂		‘There is no corner shop, or park, or whatever. Even when we do go to the park, we've got to hop in the car and go to the park’ (Middle‐Eastern parent) [35]
Lack of interesting facilities for PA		Ad; ♀				‘If you don't go here [the shopping mall in the neighbourhood], then there's nowhere else to go. Sometimes you get bored going to the same place every time you go out’ (Chinese, adolescent, female) [42]
Safety concerns		Ad; ♀, ♂	Ad; ♀, ♂	Ch, Ad; ♀, ♂	Ch; ♀, ♂	‘Around the suburb and the house, it is not safe. The park is far, because here there are people who inject drugs and they throw syringe, and if you step on it then you are finished’ (Africans parent) [45] ‘… we feel afraid to go in evenings after work to the park for our children to play or push the stroller, getting dark so soon and nobody is there…when park is empty we are feeling frightened …’ (Migrant parent) [46]
Perceived need for more equitable support/funding for sport participation			Ad; ♀, ♂			‘whether they have a … kids voucher. The thing is, everything is really expensive these days, so you can only use it for one activity and for one term. If you want to continue on … it comes at a high cost and some parents can't really afford it, so it becomes an issue then. if they extended those vouchers … to buying a bike or buying a basketball ring or buying something to get the kids moving even at home, I think that would help a lot more. I ended up buying even a second‐hand bike. Don't get me wrong, I had to buy a second‐hand bike to get them moving or I go to a second‐hand basketball ring as well to get them outside playing’ (Middle‐Eastern parent) [38]
Technology limits active travel				Ch; ♀, ♂		‘…every short distance that you need to cover, you need to be ride in a car so that is another issue. Not enough exercise, people are not exercising… But back home [Sudan], we used to travel like long distance on foot… But here [in Australia] you know, cars have taken over’ (South‐Sudanese parent) [41]
Lack of transport				Ch; ♀, ♂	Ad; ♀	‘I started to struggle [by] myself… I [was] really working very hard because I needed to help my children [with] transport…’ (South‐Sudanese parent) [41] ‘… we feel bad no transport, no car, we don't carpool with Aussie families because we cannot carpool for them, as we have no car, we cannot afford to buy a car… so children don't go to sports classes…’ (Migrant parent) [46]
*Public policy level*						
Australian policy prohibits leaving children unattended outdoors–limits outdoor PA				Ch; ♀, ♂		‘… for this country's security or laws, you don't have to leave your child alone; you need to walk with them… if you leave them outside, if these white people see them outside, they will call police for you especially those who remove children from their parents like Family SA’ (South‐Sudanese parent) [41]
Complex legislation on the use of public space limits PA participation				Ad; ♀		

*Note:* ♀, female; ♂, male; Ad, adolescent; Af, African; As, Asian; Ch, child; M‐E, Middle‐Eastern; Mi, Migrants; Mu, Muslim; PA, Physical Activity; PE, Physical Education.

## Appendix D Qualitative Results: Barriers and Facilitators of Sedentary Behaviour and Screen Time by Level of the Socio‐Ecological Model

**Table:**

	Mu	As	M‐E	Af	Mi	Example quote where provided
*Individual*						
Preference for sedentary activities			Ch; ♀, ♂			
Screen time as a stress reliever and distraction tool			Ad; ♀, ♂			
Lack of alternative activities to replace screen time			Ad; ♀, ♂			
Behaviour issues when screen time is limited			Ad; ♀, ♂			‘Yeah, no they have full withdrawals. [Child] has an emotional breakdown. And the other one, if you ask her to give her phone, she has a fit. She'll, like, run out of the room and hide in her room. The other one has withdrawals, starts shaking like he's a bloody ex junkie’ (Middle‐Eastern parent) [38]
Lack of knowledge of detrimental effects of excessive screen time			Ad; ♀, ♂			‘But I don't think they realise what effect it has on them due to it's become a norm. And because it's, become a norm, it's no longer an issue. So, it becomes other things, become mental, it's not the screen time; the screen time is an escape for them’ (Middle‐Eastern parent) [38]
Knowledge of excessive screen time engagement			Ad; ♀, ♂			
*Interpersonal*						
Screen time allowing for social interaction			Ad; ♀, ♂			‘A lot of my screen time is spent on FaceTime with my friends, especially during the holidays, and I can't really see them because they're always busy’ (Middle‐Eastern, adolescent, female) [37]
Parent knowledge of negative effects of excess screen time			Ch, Ad; ♀, ♂			‘I don't want her to just sit down and watch TV all day, it's just, you know, I don't think it's right’ (Middle‐Eastern parent) [35]
Parent awareness of excessive screen time engagement			Ad; ♀, ♂			‘They spend too much time, trust me; they binge watch everything and they're really shocking’ (Middle‐Eastern parent) [38] ‘24/7, nonstop from the second they wake up and to the second they go to sleep; even if they're in the toilet, they're on their phones. They don't get off it’ (Middle‐Eastern parent) [38]
Parent imposed screen time restrictions/rules			Ch, Ad; ♀, ♂			‘They're not allowed to have any social media. That is one; second of all, they're not allowed to watch anything while they're in their bedrooms. Have to be in the lounge room with me so I can see what they're watching … Even homework’ (Middle‐Eastern parent) [38]
*Organisational*						
Schools assist in limiting the amount of screen time			Ad; ♀, ♂			
*Community*						
Neighbourhood safety concerns lead to more screen time				Ch; ♀, ♂	Ad; ♀	‘Because we don't know what is really happening; a peaceful country, beautiful parks, but you can't see people there enjoying… This is the problem of putting children to sit just near the TV and then their eyes getting exhausted, looking at the TV instead of going to play volleyball, basketball… you get lots of evil people there’ (South‐Sudanese parent) [41] ‘…we know that playing video games means the kids don't go outside and play… maybe get lazy, but what to do I am afraid to let him play by himself outside…’ (Migrant parent) [46]
*Public Policy*						
Australian policy prohibits leaving children unattended outdoors and leads to more screen time				Ch; ♀, ♂		‘Whenever the child is indoor, he or she does not have much to do. So the only thing is to engage himself or herself in eating, sitting in front of the TV and eating’ (South‐Sudanese parent) [41]

*Note:* ♀, female; ♂, male; Ad, adolescent; Ch, child.
